# Dissection of the EIAV Core Packaging Region Identifies SL2 Stem and SL2-SL3 Junction as Gag-Associated Packaging Determinants and Antiviral Targets

**DOI:** 10.3390/ijms27114728

**Published:** 2026-05-24

**Authors:** Qiyan Chen, Rui Li, Li Wang, Jinzhong Wang, Ying Wang

**Affiliations:** 1 TEDA Institute of Biological Sciences and Biotechnology, Nankai University, 23 Hongda Street, TEDA, Tianjin 300457, China; qiyanchenn@gmail.com (Q.C.); lirui11230818@163.com (R.L.); wangli61019@163.com (L.W.); 2 Key Laboratory of Molecular Microbiology and Technology, Ministry of Education, 23 Hongda Street, TEDA, Tianjin 300457, China; 3 Tianjin Key Laboratory of Microbial Functional Genomics, 23 Hongda Street, TEDA, Tianjin 300457, China

**Keywords:** equine infectious anemia virus, packaging signal, stem-loops, RNA dimerization, antisense oligonucleotides

## Abstract

Equine infectious anemia virus (EIAV), with the simplest lentiviral genome, is a key model for studying fundamental lentiviral biology. Infectious viral particles are produced only when the Gag protein selectively encapsidates full-length genomic RNA via the packaging signal (Psi), yet the structural and functional features of EIAV Psi remain poorly characterized. Using computational prediction and dimethyl sulfate probing, we identified four stem-loops (SLs) within a ~120 nt region in the 5′ leader of the genome, spanning from downstream of the primer binding site through 20 nt into the *gag* coding sequence. In vitro dimerization assays demonstrated that a palindromic sequence (5′-CUGGCCAG-3′) within SL3 acts as a critical determinant of RNA dimerization. Functional screening using both an EIAV pseudovirus packaging system and the infectious clone EIAVuk revealed that deletion or mutation of the stem-loops significantly impairs viral packaging and replication, with SL2 deletion or its stem disruption causing the most severe defects. RNA-seq analysis of RNAs bound by wild-type Gag versus a zinc-finger mutant (H391K/H410K) identified two candidate Gag-associated sites: the SL2 stem and the SL2-SL3 junction. Targeting these regions with phosphorothioate-modified antisense oligonucleotides potently inhibited pseudovirus production and the replication of infectious EIAVuk. Our findings defined the secondary structure and functional organization of the EIAV core packaging region and established the SL2 stem and SL2-SL3 junction as candidate packaging determinants and promising targets for RNA-based antiviral intervention.

## 1. Introduction

Equine infectious anemia virus (EIAV) is a lentivirus that causes lifelong persistent infection in *Equus* species [1,2]. It possesses the smallest genome among lentiviruses, yet shares a conserved genome architecture and replication strategy with other members, including human immunodeficiency virus (HIV) [3]. Notably, EIAV is the first lentivirus for which a large-scale commercial vaccine has been developed [4]. These features make EIAV a key model for dissecting lentiviral replication and a versatile vector platform for gene delivery [5].

A critical step in the lentiviral life cycle is the selective packaging of full-length genomic RNA (gRNA) into assembling virions. Lentiviruses such as EIAV possess a dimeric, positive-sense, single-stranded RNA genome flanked by untranslated regions (UTRs) [6]. During viral assembly, the Gag protein specifically selects and encapsidates unspliced gRNA into progeny virions, which is essential for generating infectious viral particles [7,8,9,10,11]. This highly selective process is governed by specific interactions between the nucleocapsid (NC) domain of Gag and a structured *cis*-acting element in the gRNA known as the packaging signal (Psi, Ψ) [12,13,14,15].

Despite their functional importance, Psi regions across lentiviruses exhibit low conservation in primary sequences. Previous studies have established that the lentiviral Psi region, typically located within the 5′UTR, is characterized by several stem-loop (SL) structures, with the *gag* initiation codon often residing in the most 3′ SL [16]. In HIV-1, for instance, the Psi region folds into four functionally distinct SLs: SL1 harbors the dimerization initiation site (DIS) that triggers gRNA dimerization; SL2 contains the major splice donor (MSD); SL3 provides a highly conserved primary binding site for Gag; and SL4 encompasses the *gag* initiation codon [17,18,19,20]. Selective 2′-hydroxyl acylation analyzed by primer extension (SHAPE) has confirmed that the Psi regions of HIV-2 and feline immunodeficiency virus (FIV) each adopt a five-SL conformation [21,22], and predicted SL structures have also been reported for the Psi region of simian immunodeficiency virus (SIV) [23]. Mutagenesis studies further indicate that the major packaging determinants of HIV-2 and SIV are located upstream of the MSD, whereas the primary encapsidation determinant of Maedi-visna virus (MVV) resides downstream of the MSD [24,25,26].

Although the trans-activation response element (TAR) hairpin and primer binding site (PBS) of EIAV have been mapped [27,28], the functional architecture of its Psi region remains undefined. To date, only a substantially larger fragment spanning from the R region to nt 109 of the *gag* coding sequence has been shown to support packaging in an EIAV-based lentiviral vector [29]. In this study, a comprehensive structural and functional analysis of the EIAV core packaging region was performed to define features essential for genome packaging and viral replication. A ~120 nt segment within the 5′ leader was shown to fold into four SLs. Among these, the SL2 stem and SL2-SL3 junction constitute candidate Gag-associated sites, and a palindromic sequence within SL3 mediates in vitro RNA dimerization. Furthermore, targeting the SL2 stem or the SL2-SL3 junction with antisense oligonucleotides (ASOs) potently suppresses viral replication. These results establish the structural basis for EIAV genome recognition and reveal a promising RNA-targeted strategy for antiviral development against lentiviruses.

## 2. Results

### 2.1. Identification of Stem-Loop Structures in the 5′ Leader Region

To identify a candidate packaging region in EIAVuk, we applied two complementary approaches to analyze its 5′ leader sequence (nt 1–368, corresponding to the region from R to nt 109 of the *gag* coding sequence [29]). Multiple sequence alignment of EIAV isolates revealed that the region from downstream of the PBS to the vicinity of the *gag* start codon exhibits markedly elevated conservation relative to the flanking sequences (Figure S1). In parallel, RNAfold analysis [30] of the same region predicted a series of stem-loops, three of which, later termed SL1–SL3, displayed comparatively high structural confidence, whereas the flanking regions exhibited conformational flexibility (Figure S2). The convergence of high sequence conservation and high structural confidence suggested the region from downstream of the PBS to the *gag* start codon as a candidate core packaging region.

**Figure 1:**
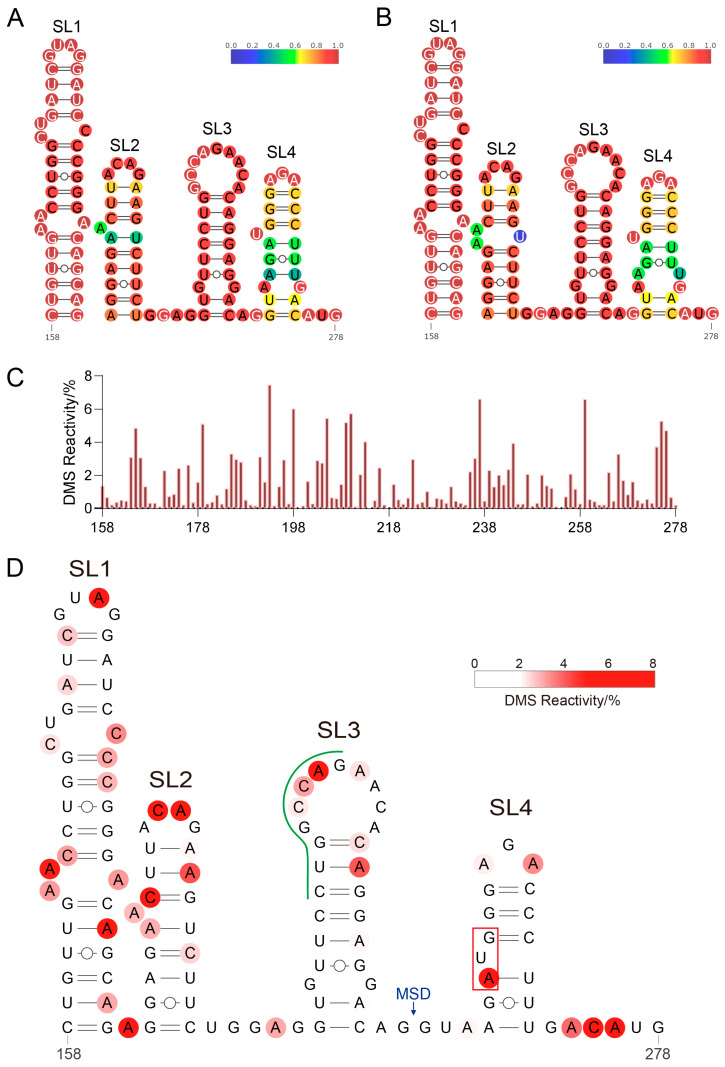
Secondary structure, and DMS reactivity of the EIAV 5′ leader region (nucleotides 158–278). (**A**) The minimal free energy secondary structure (ΔG = −39.10 kcal/mol) predicted by RNAfold. (**B**) The centroid secondary structure (ΔG = −35.50 kcal/mol) predicted by RNAfold. Nucleotides are colored according to their base pair probabilities (see key). (**C**) Quantitative DMS reactivity profile for the region. (**D**) Experimentally informed secondary structure model of the region. Sequences of four identified stem-loops (SL1–SL4) are indicated. Nucleotides are colored according to their normalized DMS reactivity (see key).

In HIV-1, the packaging signal spans from downstream of the PBS hairpin through the first 20 nt of the *gag* coding sequence and comprises four key stem-loops [18]. Guided by this organization, RNAfold prediction was performed on nt 158–278, encompassing this core region and extending 20 nt into the *gag* coding sequence. The MFE conformation (ΔG = −39.10 kcal/mol) and centroid conformation (ΔG = −35.50 kcal/mol) supported a four-stem-loop architecture, designated SL1–SL4 (Figure 1A,B). SL1 exhibited the highest structural confidence, SL2 and SL3 showed comparable stability, and SL4, which harbors the *gag* start codon, formed a more compact fold with moderate confidence.

To experimentally validate this predicted structure, DMS probing was employed, which selectively methylates unpaired adenine (A) and cytosine (C) residues at their Watson–Crick face [31]. Reverse transcription was subsequently conducted using a highly processive TGIRT enzyme (Haigene), which introduces detectable mismatches at m^1^A and m^3^C modifications [32]. The resulting mismatch-to-total ratios, which provide a quantitative measure of nucleotide accessibility at single-nucleotide resolution, were applied as soft constraints to refine the secondary-structure prediction (Figure 1C).

As expected, nucleotides located in apical loops, internal bulges, and joining regions consistently exhibited high DMS reactivity (>4%), confirming their single-stranded nature. Conversely, nucleotides within the helical stem regions showed minimal to no reactivity (<2%), which strongly supports their engagement in Watson–Crick base pairing. The DMS-constrained analysis confirmed the highly organized architecture comprising four distinct SLs, with the *gag* initiation codon (AUG) residing in the stem of SL4 and the previously reported MSD [33] located between SL3 and SL4. Notably, SL3 harbors a palindromic sequence (5′-CUGGCCAG-3′) that spans its apical loop and upper stem (Figure 1D).

In addition, sequence variations were predominantly located in apical loops and internal bulges, while stem nucleotides exhibited conservative substitutions maintaining base pairing. In SL3, for instance, a U-G pair is replaced by a U-A pair in BRA1, F2 and Liaoning strains (Figure S1), preserving stem stability. Such conservative variations at paired positions provide phylogenetic evidence that the secondary structure is functionally critical and under selective pressure.

### 2.2. The Palindromic Sequence Within SL3 Promotes RNA Dimerization

**Figure 2:**
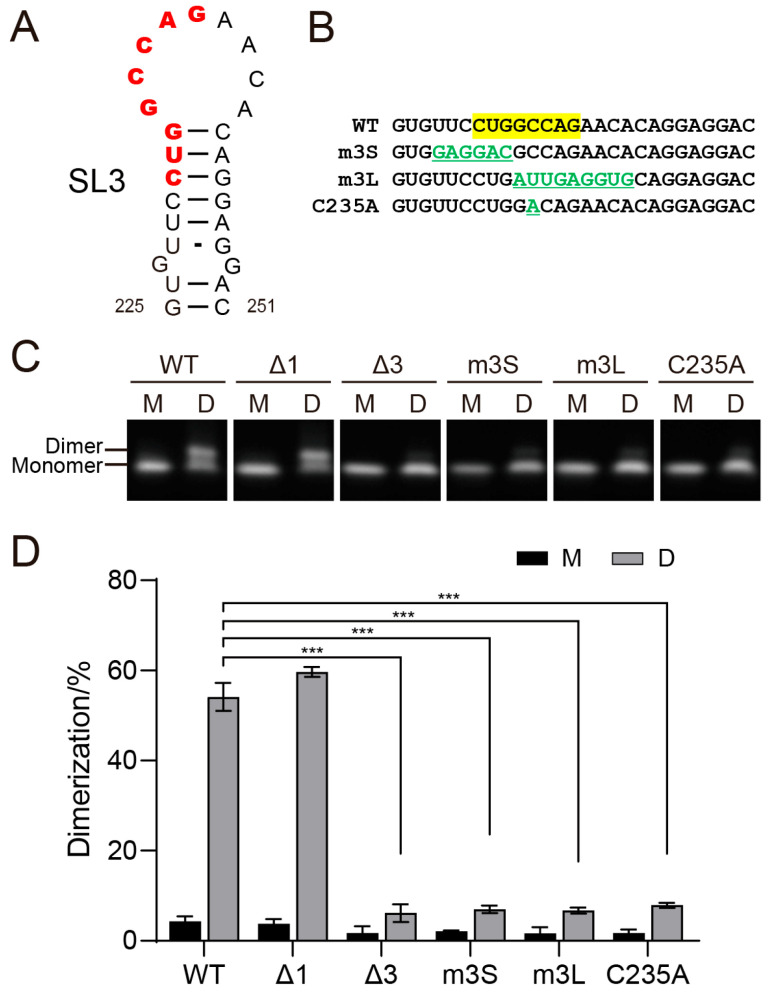
Functional analysis of the SL3 palindromic sequence in RNA dimerization. (**A**) Secondary structure of SL3, with the palindromic sequence shown in red. (**B**) Sequences of the WT SL3 and three mutants. The 8-nt palindrome is shaded in yellow, and mutated nucleotides are underlined in green. (**C**) WT and mutant RNAs were incubated in monomerization (M) or dimerization (**D**) buffer and analyzed by electrophoresis on 1% agarose gel in TBM buffer at 4 °C. (**D**) Quantification of dimerization efficiency. Dimer percentages were calculated from band intensities in (**C**) using ImageJ. Data are mean ± SD (*n* = 3). ***, *p* < 0.001.

Structural modeling revealed a conserved palindromic sequence (5′-CUGGCCAG-3′) within SL3 (Figure 2A), a hallmark of lentiviral DIS elements [34]. NUPACK simulations predicted that this palindrome mediates the most stable intermolecular interaction by refolding the SL3 upper stem and apical loop into an extended duplex (Figure S3).

To verify the predicted duplex formation, in vitro dimerization assays were conducted. In vitro-transcribed WT and mutant RNAs (nt 1–368, see Figure 2B for mutant design) were incubated under either monomer- or dimer-promoting conditions and analyzed by native agarose gel electrophoresis. Under dimer-promoting conditions, WT RNA dimerized with an efficiency of 54.11 ± 3.11%. Deletion of SL1 (Δ1) yielded a comparable level (59.66 ± 1.10%), whereas deletion of SL3 (Δ3) reduced dimerization to a near-background level (6.14 ± 1.95%). Disrupting either the SL3 helical stem (m3S) or its apical loop (m3L) severely impaired dimerization, yielding efficiencies of only 7.00 ± 0.84% and 6.70 ± 0.70%, respectively. Notably, a single C235A substitution within the palindrome markedly reduced efficiency to 7.87 ± 0.57% (Figure 2C,D). These results demonstrate that the palindromic sequence in SL3, with 3 nt in the upper stem and 5 nt in the apical loop, is essential for in vitro RNA dimerization and represents a candidate EIAV DIS element.

### 2.3. Structural Determinants of RNA Packaging and Viral Propagation

**Figure 3:**
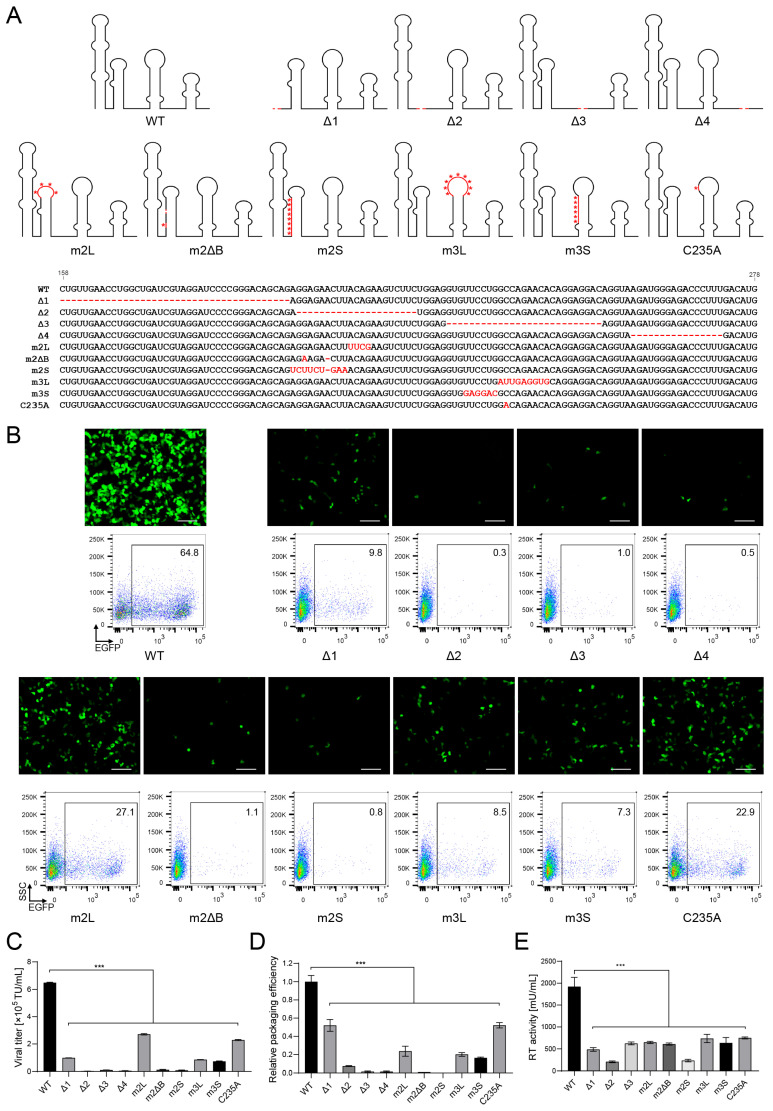
Mutational analysis of EIAV Psi structural elements in viral packaging and replication. (**A**) Schematic of WT and mutant SL1–SL4 RNA constructs. Red indicates mutations: dashed lines, deletions; asterisks, substitutions. Detailed sequences are provided in the lower part. (**B**) Pseudovirus packaging efficiency assay. HEK293T cells (2 × 10^5^) were infected with 200 μL of WT or mutant pseudovirus supernatants. EGFP expression was analyzed at 72 h post-infection. Top: Representative fluorescence microscopy images. Scale bar, 100 μm. Bottom: Flow cytometry dot plots. Boxed regions indicate EGFP-positive populations, with percentages shown. (**C**) Infectious titers of WT and Psi mutant pseudoviruses, determined from the data in (**B**). (**D**) Relative RNA packaging efficiency of wild-type and mutant viruses. Packaging efficiency was calculated as the ratio of virion-associated Psi RNA to intracellular Psi RNA, with the wild-type set as 1.0. (**E**) Replication competence of EIAVuk virions. NBL-6 cells (1 × 10^5^) were infected with virions equivalent to 500 mU of RT activity as quantified by SG-PERT. Viral replication was assessed by measuring RT activity in supernatants at 120 h post-infection. Data in (**C**–**E**) are mean ± SD (*n* = 3). ***, *p* < 0.001.

An EIAV pseudovirus packaging system was constructed by modifying a commercial three-plasmid HIV-1 vector to express EIAV components. The HIV-1 sequences in the transfer and packaging plasmids were replaced with their EIAV counterparts, and an EGFP reporter was inserted into the transfer vector to monitor packaging efficiency. Pseudoviral particles were generated by co-transfecting HEK293T cells with the transfer, packaging, and envelope plasmids. Packaging efficiency was then assessed by infecting fresh HEK293T cells with the harvested supernatants and quantifying EGFP expression via fluorescence microscopy and flow cytometry (Figure 3A–C). The WT EIAV Psi construct supported robust infectious pseudovirus production, yielding 64.8% EGFP-positive cells (Figure 3B), which corresponded to a functional titer of (6.48 ± 0.04) × 10^5^ TU/mL (Figure 3C). Deletion of individual SLs caused severe titer reductions: deletion of SL1 (Δ1), SL2 (Δ2), SL3 (Δ3), or SL4 (Δ4) decreased titers to (0.98 ± 0.01) × 10^5^, (0.03 ± 0.01) × 10^5^, (0.10 ± 0.01) × 10^5^, and (0.05 ± 0.01) × 10^5^ TU/mL, respectively. We next examined specific structural elements within SL2 and SL3. In SL2, substitution of the purine-rich loop with a stable UUCG tetraloop (m2L) decreased the titer to (2.71 ± 0.05) × 10^5^ TU/mL, whereas disruption of the stem (m2S) or alteration of the stem by bulge elimination and a substitution (m2ΔB) caused more severe defects, reducing titers to (0.08 ± 0.01) × 10^5^ and (0.11 ± 0.03) × 10^5^ TU/mL, respectively. Mutations targeting the loop (m3L) or stem (m3S) of SL3 reduced titers to (0.85 ± 0.01) × 10^5^ and (0.73 ± 0.03) × 10^5^ TU/mL. Notably, the dimerization-deficient mutant (C235A) exhibited a less severe reduction to (2.29 ± 0.04) × 10^5^ TU/mL (Figure 3A–C). Intracellular Gag protein levels in producer cells showed modest changes (0.89- to 1.26-fold relative to WT), whereas Psi RNA exhibited more pronounced variation (0.26- to 2.27-fold relative to WT). Even after normalization to Psi RNA levels, severe titer defects persisted (Figure S4), indicating that impaired packaging efficiency, rather than limited RNA availability, accounts for these defects.

To directly validate this, we quantified the relative packaging efficiency, defined as the ratio of virion-associated to intracellular Psi RNA (Figure 3D). Consistent with the pseudoviral titer assays, the relative packaging efficiencies of the mutants closely correlated with their infectivity defects, confirming that the targeted structural elements within the Psi region are indispensable for packaging genomic RNA into virions.

The infectious clone EIAVuk and the replication-permissive NBL-6 cells were employed to evaluate the impact of SL deletions and mutations in the context of the complete viral life cycle. HEK293T-derived WT and mutant virions, normalized to equal RT activity, were used to infect NBL-6 cells. WT virus replicated efficiently, yielding 1921.8 ± 211.9 mU/mL RT activity at 120 h post-infection. Consistent with the pseudovirus packaging results, all tested variants exhibited significantly reduced replication capacity. Among them, the Δ2 and m2S mutants showed the most severe defects, with RT activity reduced to 204.3 ± 19.5 and 232.3 ± 29.0 mU/mL, respectively. Other mutants displayed less severe yet substantial impairment, with RT activity maintained at approximately 600 mU/mL (Figure 3E). The Δ4 mutant was excluded from this assay because SL4 contains the *gag* initiation codon, which is essential for Gag synthesis and viral particle production.

### 2.4. RNA-Seq Identifies the SL2 Stem and SL2-SL3 Junction as Candidate Gag-Associated Sites

**Figure 4:**
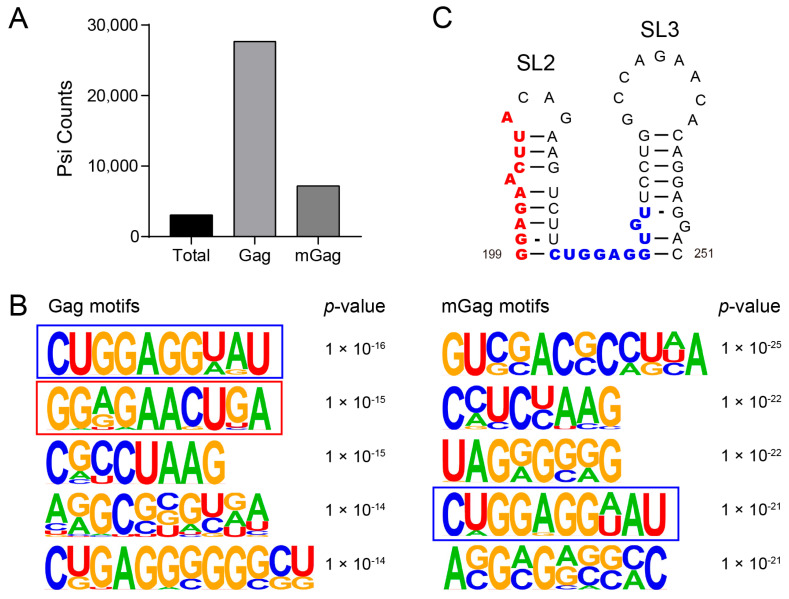
RNA-seq analysis of RNAs bound by WT and H391K/H410K mutant Gag. (**A**) Read counts mapping to the Psi region in input (Total), WT Gag-bound (Gag), and mutant Gag-bound (mGag) samples. Total, total RNA from *E. coli* expressing the Psi transcript; Gag and mGag, RNAs extracted from Gag-RNA and mGag-RNA complexes, respectively. (**B**) Enriched RNA motifs identified by HOMER [36]. Top 5 motifs in Gag and mGag samples are shown. Motif 1 (Gag) and motif 4 (mGag) are boxed in blue; motif 2 (Gag) is boxed in red. *p*-values indicate statistical significance of motif enrichment. (**C**) Secondary structure of SL2 and SL3. The SL2-SL3 junction (matching Gag motif 1 and mGag motif 4) is colored in blue. The 5′ arm of the SL2 stem (matching Gag motif 2) is colored in red.

The selective packaging of lentiviral gRNA is critically dependent on the interaction between the NC domain of the Gag protein and the Psi element within the gRNA [14,35]. To identify RNA sequences preferentially bound by EIAV Gag, we purified His-tagged protein-RNA complexes from *E. coli* expressing Gag or mutant Gag (mGag, Gag with H391K and H410K substitutions in the zinc-finger domains) with EIAV Psi (Figure S5). RNA was extracted from the complexes and from total cell lysates (input) for high-throughput sequencing. Comparative analysis revealed that sequencing reads mapping to the Psi region were significantly enriched in Gag-bound samples compared to both the mGag control and the total RNA input (Figure 4A).

MACS2 peak calling on reads aligned to the *E. coli* genome identified 290 and 60 regions significantly enriched in the Gag and mGag samples, respectively. De novo motif discovery was subsequently performed on these peaks using HOMER, and the top five enriched motifs for Gag and mGag are shown in Figure 4B. Gag motif 1 was also detected in the mGag control (Figure 4B, blue box), indicating RNA binding independent of the canonical NC domain zinc fingers. Further analysis revealed that this motif matches the SL2-SL3 junction of the core packaging region (Figure 4C). Notably, Gag motif 2 (Figure 4B, red box) was exclusively enriched in the Gag-bound RNA and aligned perfectly with the 5′ arm of the SL2 stem (Figure 4C). These results establish the SL2 stem and the SL2-SL3 junction as two candidate Gag-associated sites.

To determine whether the identified Gag-associated sites also contribute to RNA structural organization, we introduced mutations into the SL2 stem (2Smut) and the SL2-SL3 junction (23Jmut) and assessed their effects on Psi RNA dimerization in vitro. Disruption of either motif reduced dimer formation (Figure S6), suggesting that these elements play a dual role in both Gag binding and RNA dimerization.

To further dissect the contribution of primary sequence versus secondary structure within the SL2 stem, we generated a compensatory mutation that restored the predicted base-pairing while altering the nucleotide sequence (2CM). Compensatory mutants still showed a significant decrease in viral titer compared to wild-type Psi (Figure S7), indicating that the SL2 stem function depends on both its structural integrity and specific sequence identity. Collectively, these results suggest that Gag recognition of the SL2 region involves sequence-specific contacts within a defined structural context.

### 2.5. ASOs Targeting SL2 Stem or SL2-SL3 Junction Inhibit Viral Packaging and Propagation

Having identified two candidate Gag-associated sites, we next asked whether sequestering these regions could inhibit viral assembly and spread. To this end, we designed phosphorothioate-modified ASOs targeting the SL2 stem and the SL2-SL3 junction, named ASO-2S and ASO-23J, respectively, and evaluated their efficacy in an ASO antiviral assay [37].

**Figure 5:**
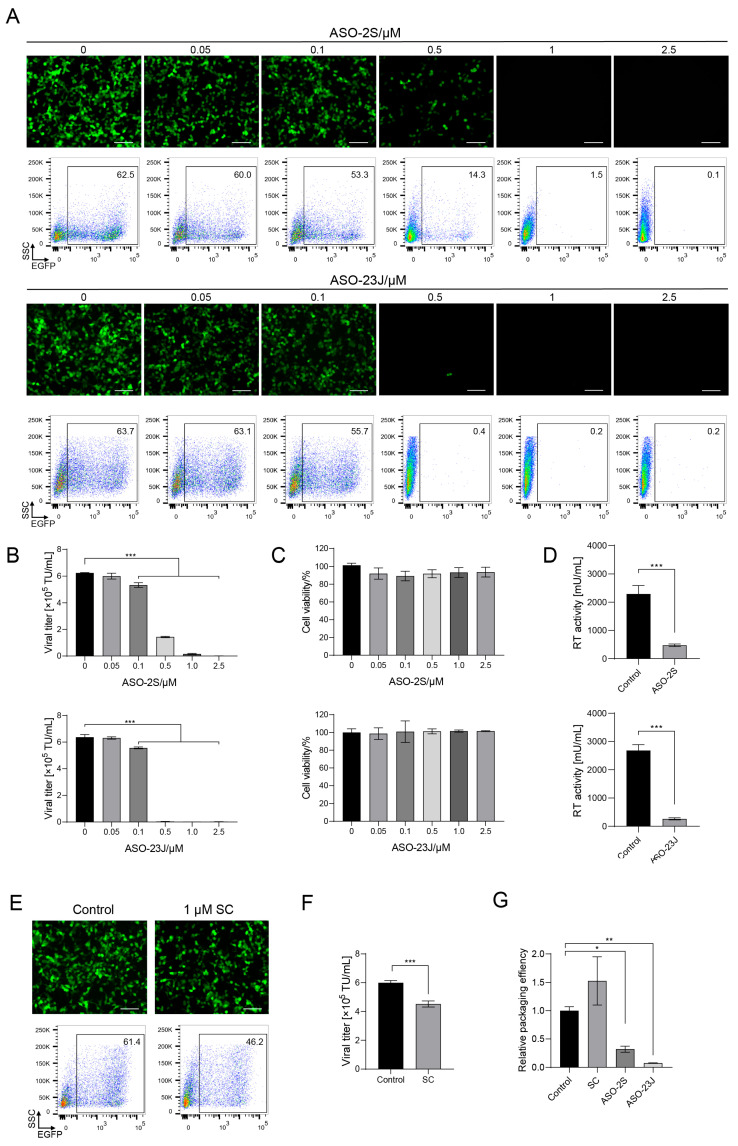
Effects of ASO-2S and ASO-23J on viral packaging and replication. (**A**) Inhibition of pseudovirus production by ASO-2S or ASO-23J. Pseudoviruses were produced in the presence of the indicated ASO concentrations, and HEK293T cells (2 × 10^5^) were infected with 200 μL of supernatant. EGFP expression was analyzed at 72 h post-infection. Top: Representative fluorescence microscopy images. Scale bar, 100 μm. Bottom: Flow cytometry dot plots. Boxed regions indicate EGFP-positive populations (percentages shown). (**B**) Dose-dependent inhibition of infectious pseudoviral titers, derived from the data in (**A**). (**C**) Cytotoxicity of ASO-2S and ASO-23J in HEK293T cells. Cell viability was assessed by CCK-8 assay after 48 h of ASO exposure. (**D**) Inhibition of replication-competent EIAVuk by ASO-2S or ASO-23J. NBL-6 cells (1 × 10^5^) were infected with EIAVuk (500 mU RT activity) and treated with 1 μM ASO-2S or ASO-23J. Viral replication was quantified by RT activity in supernatants at 120 h post-infection. (**E**) Effect of 1 μM SC on pseudovirus production. (**F**) Detection of infectious pseudoviral titers, derived from the data in (**E**). (**G**) Relative RNA packaging efficiency in wild-type pseudovirus-producing cells treated with 1 μM ASO-2S, ASO-23J or SC. Packaging efficiency was calculated as the ratio of virion-associated Psi RNA to intracellular Psi RNA, with the wild-type control set as 1.0. Data are mean ± SD (*n* = 3). *, 0.01 < *p* < 0.05; **, 0.001 < *p* < 0.01; ***, *p* < 0.001.

When added to producer cells during pseudovirus packaging, ASO-2S reduced particle release in a dose-dependent manner, nearly abolishing production at concentrations of 1 μM and above (Figure 5A,B), without significantly affecting cell viability (Figure 5C). ASO-23J exhibited a similar dose-dependent inhibition, virtually eliminating production at 0.5 μM and above (Figure 5A,B), without compromising cell viability (Figure 5C).

We further examined the effect of these ASOs on spreading infection. EIAVuk-infected NBL-6 cells treated with 1 μM ASO-2S showed significantly suppressed viral propagation, with RT activity reduced from 2291.1 ± 299.9 to 473.3 ± 45.4 mU/mL at 120 h post-infection. Similarly, ASO-23J treatment at 1 μM potently suppressed viral replication, reducing RT activity from 2684.8 ± 207.6 to 264.6 ± 37.5 mU/mL by day 5 (Figure 5D). In both cases, cell viability remained above 80%.

To assess sequence specificity, we included a scrambled phosphorothioate ASO (SC) as a control. Treatment with 1 μM SC reduced viral titer from (6.00 ± 0.16) × 10^5^ to (4.52 ± 0.21) × 10^5^ TU/mL with cell viability remaining above 80% (Figure 5E,F). In contrast, ASO-2S and ASO-23J reduced viral titer to near-background levels at the same concentration, demonstrating substantially greater inhibitory activity than SC.

To investigate the underlying mechanism, we quantified intracellular viral RNA and Gag protein levels in ASO-treated producer cells. ASO treatment reduced Psi RNA levels to approximately 50% of untreated controls, whereas Gag protein expression remained unaffected (Figure S8A,B). Even when viral titer was normalized to intracellular RNA levels, ASO-treated samples still exhibited a significant reduction compared to controls (Figure S8C). Furthermore, 1 μM ASO-2S and ASO-23J significantly decreased genomic RNA packaging efficiency relative to the untreated control, whereas SC had no such effect (Figure 5G), confirming that the antiviral activity is sequence-specific.

## 3. Discussion

In this study, we characterized the secondary structure of the EIAV core packaging region using an integrative approach that combined computational prediction, chemical probing, and mutational analysis. Our work defined a structural element that begins downstream of the PBS and extends 20 nt into the *gag* coding sequence, folding into four distinct SLs. The application of DMS chemical probing significantly refined the structural model of this critical region, resolving ambiguities inherent in computational predictions alone. For instance, although thermodynamic modeling initially suggested alternative base-pairing possibilities at the base of SL4, the consistently high DMS reactivity observed in this region confirms its single-stranded state in our experimental context.

We identified a conserved palindromic sequence within SL3 as a putative DIS. In lentiviruses, the DIS location and structural context vary considerably: in HIV-1, HIV-2, and SIV, the DIS is located in the loop of SL1; in FIV, it spans the entire SL5; and in MVV, it resides within SL2 [21,38,39,40]. In some retroviruses, the DIS spans portions of both the stem and loop [34]. Notably, this palindromic sequence identified in EIAV is identical to the core of the well-characterized PAL1 dimerization motif in Moloney murine leukemia virus, both in nucleotide identity and positional arrangement [41]. Retroviral genome packaging is generally associated with RNA dimerization mediated by such motifs [34]. However, our mutational analysis reveals that although this motif is essential for dimerization, its disruption does not abolish genome packaging, suggesting that the overall structural scaffold of SL3 contributes more critically to packaging than the specific base pairing at the palindromic interface. Additionally, other genomic regions or protein-mediated bridging may partially compensate for impaired dimerization to maintain packaging efficiency.

The 5′ arm of the SL2 stem and the SL2-SL3 junction are essential and specific structural determinants for Gag recruitment and genome packaging in EIAV. The requirement for the SL2 stem suggests recognition of a specific helical geometry by Gag. Disruption of the SL2 stem severely reduced viral titer (Figure 3), and compensatory mutations that restore base-pairing while altering the primary sequence failed to rescue this defect. Although alternative RNA misfolding of the compensatory mutant construct cannot be completely excluded, these findings demonstrate that both structural integrity and specific nucleotide identity are required for SL2 function. This is analogous to HIV-1, where NC recognition of SL3 is mediated by specific contacts between the zinc knuckles and exposed guanosines within a defined stem-loop architecture [17]. Meanwhile, the enrichment of unpaired guanosines within the SL2-SL3 junction mirrors the specific NC-guanine interactions observed in HIV-1 and is reminiscent of the purine-rich linkers in Mason-Pfizer Monkey Virus [42,43]. Furthermore, mutations at either the SL2 stem or the SL2-SL3 junction reduced Psi RNA dimerization in vitro, revealing that these Gag-associated sites are involved in higher-order RNA structural organization. Unlike HIV-1, where the primary encapsidation determinants are centered on SL3 [17], or MVV, where determinants reside downstream of the MSD but involve different structural elements [26], EIAV Gag appears to preferentially engage regions upstream of the MSD, namely the SL2 stem and the SL2-SL3 junction. This distinct recognition mechanism underscores how different lentiviruses have evolved divergent structural solutions to achieve the conserved functional outcome of specific genome encapsidation.

The dynamic nature of lentiviral 5′ leaders allows the RNA to toggle between alternative structural conformations competent for either translation or packaging, thereby regulating distinct stages of the viral life cycle. Such functional switching has been reported in HIV-1, where 5′UTR rearrangements are predicted to favor or inhibit packaging [11]. A similar model has been proposed for FIV, where long-range interactions and multiple stem-loop conformations are invoked to modulate the exposure of either the DIS or the *gag* start codon [21]. Our structural data suggest a comparable regulatory mechanism, although direct evidence is lacking. One possibility is that the EIAV leader could adopt an equilibrium between two functional states potentially mediated by the U5 region (Figure S9). In one conformation, U5 sequestration of the *gag* start codon AUG would expose the palindromic DIS in SL3 for RNA dimerization, potentially favoring packaging. Alternatively, U5 engagement of the DIS might release the *gag* start codon to facilitate ribosome entry and translation. This speculative model involves an alternative PBS hairpin conformation distinct from that reported previously [28]. Should this hypothesis prove relevant, future cryo-EM or NMR studies could help visualize these dynamic rearrangements.

To dissect the functional roles of individual structural elements, we employed both the single-round pseudovirus system and the replication-competent EIAVuk system. The pseudovirus system isolates *cis*-acting Psi function from potential confounding effects on Gag protein expression or function, ensuring that observed defects directly reflect packaging efficiency. However, this assay captures only the initial packaging and a single infection cycle. In contrast, the replication-competent EIAVuk system enables the assessment of mutations throughout the full viral life cycle, although it cannot accommodate mutations that disrupt overlapping essential features. This is exemplified by SL4, which was not targeted for deletion in EIAVuk because it contains the *gag* start codon. Importantly, the results from both systems were highly concordant: mutations that severely diminished pseudovirus production (e.g., Δ2 and m2S) caused corresponding defects in viral propagation in permissive NBL-6 cells. This strong correlation confirms that the observed replication defects are predominantly attributed to impaired packaging and validates the pseudovirus system as a reliable tool for mapping EIAV packaging determinants.

Identifying the SL2 stem and SL2-SL3 junction as Gag-dependent packaging determinants establishes them as candidate therapeutic targets. Our data demonstrate that ASO-mediated targeting of these conserved regions can suppress viral propagation. The reduction in intracellular Psi RNA without affecting Gag protein levels suggests RNase H-mediated degradation of the target RNA [44]. However, viral titer remained significantly reduced even after normalization to intracellular RNA levels, and packaging efficiency was impaired exclusively in ASO-treated cells but not in SC-treated control, indicating that the ASOs also block genome packaging through a sequence-specific mechanism. Although SC exerted a modest antiviral effect, the sequence-specific ASOs achieved near-complete suppression of virus production, highlighting the functional importance of these sites. This approach aligns with the emerging paradigm of structure-guided antivirals, which exploit essential RNA structures for intervention, as recently evidenced against HIV-1 and SARS-CoV-2 [37,45]. Future biochemical and structural studies, such as competition EMSA or NMR analysis of the Gag–Psi complex, will be needed to elucidate the molecular details of this interaction. Collectively, our study defines the structural determinants of EIAV genome packaging and identifies a functionally critical RNA element, providing a foundation for the development of RNA-targeted antiviral strategies.

## 4. Materials and Methods

### 4.1. Plasmids

The lentiviral packaging plasmid psPAX2 and envelope plasmid pMD2.G were obtained from MiaoLingBio, Wuhan, China. The EIAV packaging plasmid psPAX2-EIAVGagpol was constructed by replacing the HIV-1 sequence in psPAX2 with EIAV *gag-pol* and *rev*-*s2* from EIAVuk [46,47]. The resulting EIAV vectors are replication-defective, with essential viral elements split across separate plasmids, minimizing the risk of recombination. The EIAV transfer vector pEIAV was engineered by inserting the CMV promoter and essential *cis*-acting elements into the pHAGE backbone. These elements, including EIAV RU5, cPPT, RRE, and 3′LTR, were obtained either by PCR using primers listed in Table S1 or by gene synthesis (Azenta Life Sciences, Suzhou, China), and subsequently incorporated into the vector by overlap PCR-based cloning strategy [48]. The reporter construct pEIAV-CEGFP was created by inserting a PCR-amplified CMV-EGFP expression cassette into pEIAV.

To generate mutants in both the EIAVuk infectious clone and the pEIAV-CEGFP transfer vector, the Psi region was mutagenized via inverse PCR. Briefly, mutagenesis was performed on shuttle vectors using primers flanking the target site and incorporating the desired mutations (Table S1). Following amplification, the PCR products were DpnI-treated, circularized and subcloned into the infectious clone and transfer vector, respectively.

To generate RNAs for in vitro dimerization assays, transcription templates were constructed by cloning the 5′ leader region of EIAVuk into the pBluescript II SK(+) vector. Mutations within these regions were introduced using the inverse PCR-based mutagenesis strategy described above.

For RNA-seq analysis, three plasmids were prepared. Plasmid pET28b-EIAV Gag, which expresses WT EIAV Gag in *E. coli*, was described previously [49]. A gene fragment encoding mGag (Gag with H391K and H410K substitutions in the zinc-finger domains) was synthesized and inserted into the pET28b vector. The Psi region was cloned into pBluescript II SK(+) to generate pBS-Psi, using primers listed in Table S1.

All plasmids constructed in this study (Table S2) were verified by sequence analysis.

### 4.2. Cell Culture, Transfection, and Infection

HEK293T cells and NBL-6 equine dermal cells (ATCC^®^ CCL-57™) were maintained at 37 °C with 5% CO_2_ in Dulbecco′s modified Eagle′s medium (DMEM) supplemented with 10% (*v*/*v*) fetal bovine serum, 100 U/mL penicillin, and 100 μg/mL streptomycin. HEK293T is a commercially available immortalized cell line, no new primary human tissue was used in this study. Transfection was performed with Hieff Trans^®^ PEI 40,000 (Yeasen, Shanghai, China) according to the manufacturer′s protocol. To generate pseudovirus particles, HEK293T cells were co-transfected with pEIAV-CEGFP or the indicated mutant, together with pMD2.G and psPAX2-EIAVGagpol. Infectious EIAVuk virions were generated by transfecting HEK293T cells with the EIAVuk infectious clone or its derivatives. At 72 h post-transfection, virus-containing supernatant was harvested, clarified by centrifugation at 1000× *g* for 10 min, and filtered through a 0.45 μm membrane. For infection, HEK293T cells were challenged with 200 μL of pseudovirus-containing supernatant, and NBL-6 cells were infected with equivalent amounts of WT or mutant EIAVuk supernatant normalized by reverse transcriptase (RT) activity.

All procedures involving infectious materials were performed in a Class II biosafety cabinet under Biosafety Level 2 (BSL-2) containment. EIAV does not infect humans. Waste was handled per institutional and BSL-2 guidelines.

### 4.3. RNA Secondary Structure Prediction

The 5′ leader sequence of EIAVuk (nt 1–368, from R to nt 109 of the *gag* coding sequence) was analyzed by multiple sequence alignment of EIAV isolates using ClustalW [50] and by RNA secondary structure prediction using RNAfold [30]. For focused structural analysis of nt 158–278, the MFE and centroid structures were generated using RNAfold. RNA dimerization was simulated using NUPACK [51].

### 4.4. Dimethyl Sulfate (DMS) Modification and Sequencing

For DMS probing, HEK293T cells transfected with EIAVuk were incubated without or with 2% (*v*/*v*) DMS in DMEM at 37 °C for 5 min [31,52], then quenched with 30% (*v*/*v*) β-mercaptoethanol. Total RNA was extracted using TRIzon (CWBio, Taizhou, China), and reverse transcription was performed with TGIRT-III Reverse Transcriptase (Haigene, Harbin, China) according to the manufacturer′s instructions. The reaction was terminated by adding 1/20 volume of 5 M NaOH, followed by incubation at 95 °C for 2 min and neutralization with 1/20 volume of 5 M HCl. Using cDNA as the template, PCR was performed with DMS modification primers (Table S1). Samples were submitted to CWBio Biotech Co., Ltd. for nanopore sequencing to determine per-base mutation frequencies. DMS reactivity profiles were generated and mapped onto the RNA structure model using VARNA [53].

### 4.5. In Vitro Transcription and RNA Dimerization Assay

DNA templates for in vitro transcription were generated by PCR amplification using primers listed in Table S1 and purified via agarose gel electrophoresis and extraction. In vitro transcription was performed using the purified templates with the T7 High Yield RNA Synthesis Kit (Sangon Biotech, Shanghai, China) according to the manufacturer′s protocol. Reactions were incubated at 37 °C for 4 h and subsequently treated with DNase I to remove template DNA. The synthesized RNA was purified by lithium chloride precipitation and stored at −80 °C until use.

In vitro dimerization of WT and mutant RNAs was assessed as described [54], with modifications. Briefly, 3 pmol of RNA was incubated at 37 °C for 30 min in either dimerization buffer (50 mM sodium cacodylate, pH 7.5, 300 mM KCl, 2 mM MgCl_2_) or monomerization buffer (50 mM sodium cacodylate, pH 7.5, 40 mM KCl, 0.1 mM MgCl_2_). RNAs were then resolved by 1% native agarose gel electrophoresis in TBM buffer (50 mM Tris, pH 8.3, 45 mM boric acid, 0.1 mM MgCl_2_) at 110 V and 4 °C. Gels were stained with 0.5 μg/mL ethidium bromide, and bands were visualized under UV transillumination. The intensities of dimeric and monomeric bands were quantified using ImageJ software (version 1.8.0), and the dimerization percentage was calculated.

### 4.6. Fluorescence Imaging

At 72 h post-infection, HEK293T cells were imaged with an Axio Observer 7 inverted microscope (Carl Zeiss, Jena, Germany) equipped with a 20× objective. Imaging parameters, including exposure time and gain, were kept consistent across all samples. EGFP fluorescence was captured using 495 nm excitation and 519 nm emission filters. Images were acquired and processed with ZEN 3.9 software.

### 4.7. Flow Cytometry Analysis

For flow cytometry analysis, HEK293T cells were harvested at 72 h post-infection. Samples were analyzed on a FACSymphony™ A5 flow cytometer (BD Biosciences, San Jose, CA, USA). EGFP fluorescence was detected using the FITC channel. The percentage of EGFP-positive cells was determined by gating so that <0.1% of uninfected control cells fell within the positive region. Functional viral titers (TU/mL) were calculated using the formula: (number of cells at infection × percentage of EGFP-positive cells)/volume of virus inoculum (mL) [55].

### 4.8. Packaging Efficiency Assay

Virion-associated and intracellular RNAs were extracted at 48 h post-transfection using the E.Z.N.A.^®^ Viral RNA Kit (Omega Bio-tek, Norcros, GA, USA) for virions or TRIzon reagent (CWbio) for cells, respectively, treated with DNase I, reverse-transcribed, and quantified by qPCR on a QuantStudio™ 5 Real-Time PCR System (Thermo Fisher Scientific, Waltham, MA, USA). Packaging efficiency was calculated as the ratio of virion associated with intracellular Psi RNA [56].

### 4.9. SYBR Green I-Based Product-Enhanced RT (SG-PERT) Assay

Viral particle concentration in culture supernatant was quantified by measuring RT activity using the SG-PERT assay as described [57]. Reactions were performed on a QuantStudio™ 5 Real-Time PCR System, with an initial reverse transcription step at 42 °C for 20 min, followed by standard qPCR cycling. Quantification cycle values were converted to absolute RT activity units by interpolation from a standard curve generated using serial dilutions of Moloney murine leukemia virus RT (Takara Bio, Dalian, China).

### 4.10. Cell Viability Assay

The cytotoxicity of the ASOs was evaluated using the Cell Counting Kit-8 (CCK-8, Beyotime, Shanghai, China) according to the manufacturer′s instructions. Briefly, cells (5 × 10^3^ cells/well) were seeded in 96-well plates and cultured for 24 h to allow adhesion. Cells was subsequently treated with serially diluted ASOs for 48 h (HEK293T) or 60 h (NBL-6). After treatment, 10 µL of CCK-8 reagent was added to each well, and the plates were incubated at 37 °C for 1.5 h. Absorbance at 450 nm was measured using a Spark^®^ Multimode Microplate Reader (Tecan, Männedorf, Switzerland). Cell viability was normalized to mock-treated cells.

### 4.11. His-Affinity Purification of RNA-Protein Complexes Followed by RNA-Seq

*E. coli* BL21 (DE3) cells were co-transformed with pBS-Psi and either pET28b-EIAV Gag or pET28b-EIAV mGag (H391K/H410K). Following induction with 0.1 mM isopropyl β-D-1-thiogalactopyranoside at 26 °C for 4 h, cells were harvested and His-tagged protein-RNA complexes were purified by His-affinity chromatography as described [58]. RNA was then extracted from purified complexes and from total cell lysates (input), and samples were submitted to Azenta Life Sciences for high-throughput sequencing on an Illumina HiSeq 2000 platform. For data analysis, adapter sequences and low-quality bases were trimmed using Cutadapt [59]. Clean reads were aligned to the *E. coli* reference genome using Bowtie2 [60]. MACS2 peak calling was performed to identify enriched peaks in the pull-down samples relative to the input control [61]. De novo motif discovery within enriched peak regions was performed using HOMER [36].

### 4.12. ASO Antiviral Assays

Phosphorothioate-modified ASOs targeting the SL2 stem (ASO-2S: 5′-TGTAAGTTCTCCTCTGCTGT-3′) or the SL2-SL3 junction (ASO-23J: 5′-CAGGAACACCTCCAGAAGAC-3′) and scrambled phosphorothioate-modified ASO (SC: 5′-CGTCGTTGTTTCGACTCTAT-3′) were synthesized by Azenta Life Sciences.

For the pseudovirus system, HEK293T cells were seeded at a density of 2 × 10^5^ cells per well and infected with 200 μL of pseudovirus-containing supernatant. At 3 h post-transfection, ASO was added to the culture medium at the indicated concentrations. EGFP expression was analyzed at 72 h post-infection using fluorescence microscopy and flow cytometry.

For the replication-competent EIAVuk system, NBL-6 cells were seeded at a density of 1 × 10^5^ cells per well and infected with EIAVuk for 12 h. Following infection, cells were washed with phosphate-buffered saline to remove unbound virus, and fresh medium containing ASO was added. The culture medium was replenished with fresh medium containing ASO at 60 h post-infection to maintain effective concentrations. Viral replication was assessed at 120 h post-infection by the SG-PERT assay.

### 4.13. Statistical Analysis

Statistical significance was assessed using one-way analysis of variance (ANOVA) followed by Dunnett′s multiple comparisons test or an unpaired, two-tailed Student′s *t*-test. A *p*-value of less than 0.05 was considered statistically significant.

## Data Availability

All study data are included in the article or the Supplementary Information File. The RNA-seq data are available from NCBI SRA database under the accession number SRA1423464.

## Supplementary Materials

Supporting information can be downloaded at: https://www.mdpi.com/article/10.3390/ijms27114728/s1.

## Abbreviations

The following abbreviations are used in this manuscript:

EIAV	Equine infectious anemia virus
Psi	Packaging signal
SL	Stem-loop
gRNA	Genome RNA
UTR	Untranslated region
DIS	Dimerization initiation site
MSD	Major splice donor
PBS	Primer binding site
ASO	Antisense oligonucleotide
DMS	Dimethyl sulfate
