# Supplementary material for: Dissection of the EIAV Core Packaging Region Identifies SL2 Stem and SL2-SL3 Junction as Gag-Associated Packaging Determinants and Antiviral Targets
Source: Int J Mol Sci. 2026 May 24;27(11):4728. doi: 10.3390/ijms27114728 (PMC13257266; doi:10.3390/ijms27114728)
Supplement: Supplementary file 1 [file ijms-27-04728-s001.zip › ijms-4275254-supplementary.pdf]

Supporting Information File

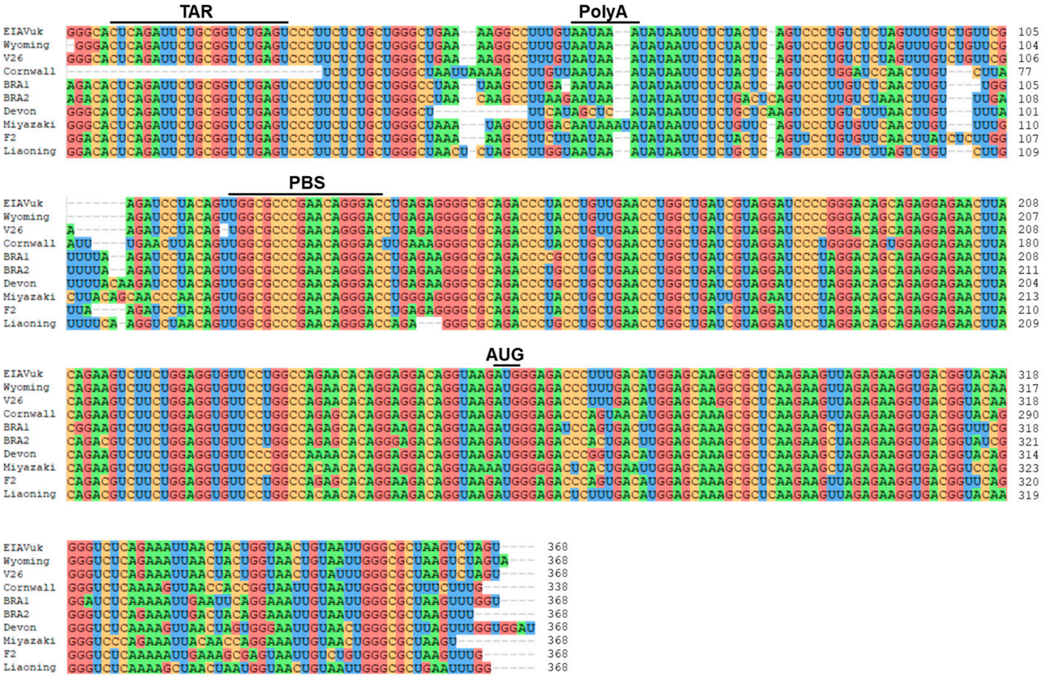

Figure S1. Multiple sequence alignment of nt 1–368 of the 5′ leader across ten replication-competent EIAV strains. The TAR, polyA, PBS, and the *gag* AUG are annotated. Numbers indicate genomic positions in the reference genome RNA. Strain names and GenBank accession numbers: EIAVuk (AF016316.1), Wyoming (AF033820.1), V26 (AB008197.1), Cornwall (MH580898.1), BRA1 (MN560970.1), BRA2 (MN560971.1), Devon (MH580897.1), Miyazaki (JX003263.1), F2 (JX480631.1), Liaoning (AF327877.1).

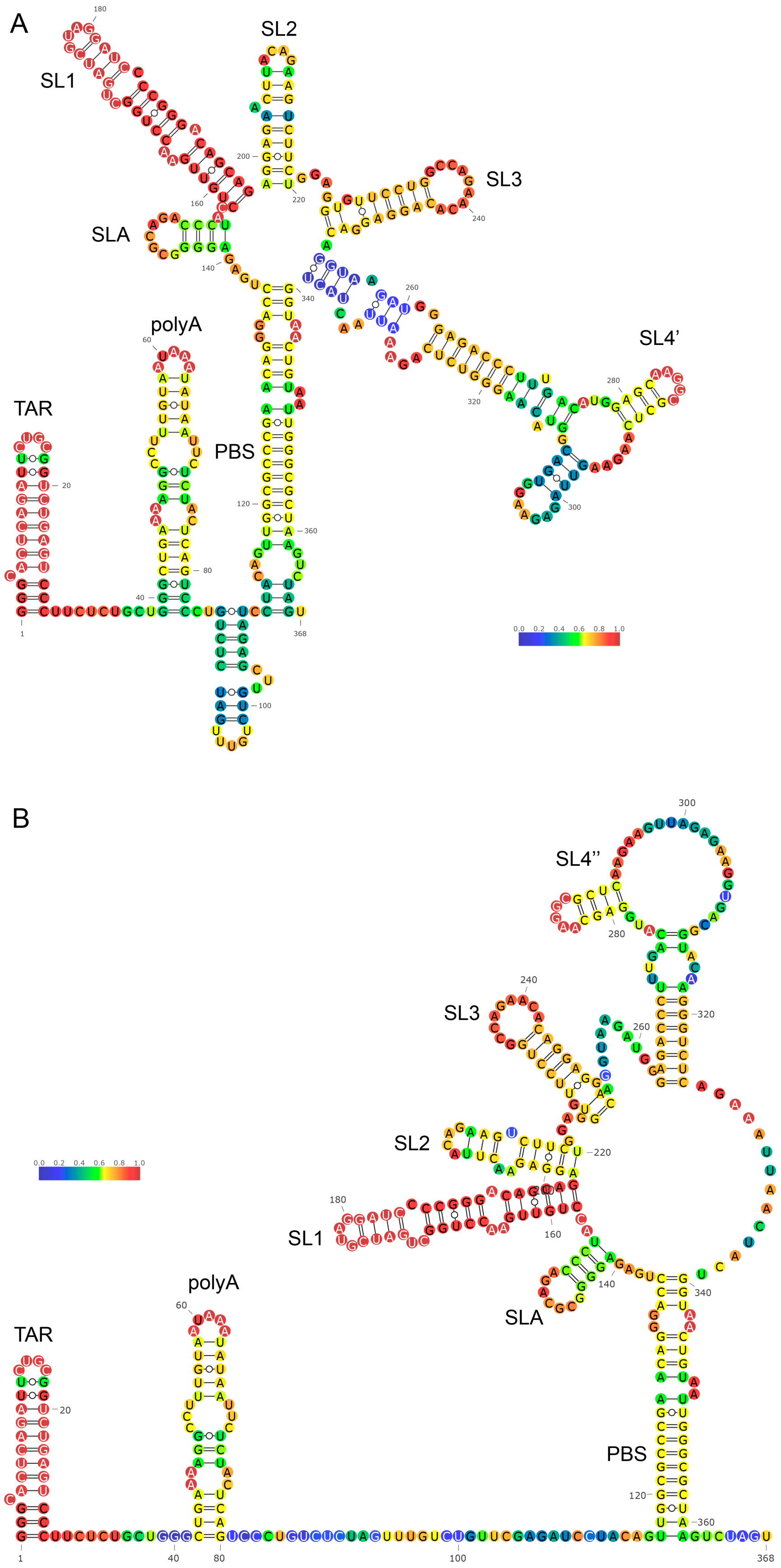

Figure S2. The secondary structure of nt 1–368 of EIAVuk gRNA predicted by RNAfold. (A) The minimal free energy structure (ΔG = -126.30 kcal/mol). (B) The centroid structure (ΔG = -106.90 kcal/mol). Numbers indicate genomic positions in the reference genome RNA. Nucleotides are colored according to their base pair probabilities (see key).

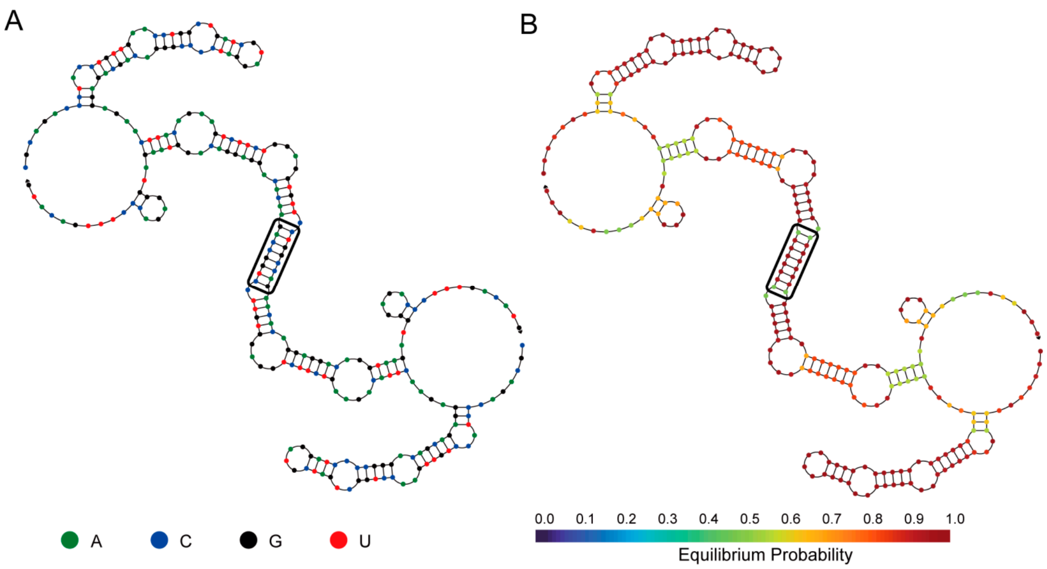

Figure S3. NUPACK-predicted homodimeric MFE proxy structure of the 146–278 nt of EIAVuk gRNA. (A) Nucleotide identity shading (A=green, C=blue, G=black, U=red). (B) Equilibrium probability shading for the depicted dimeric state. The palindromic sequence facilitating dimerization is boxed in black.

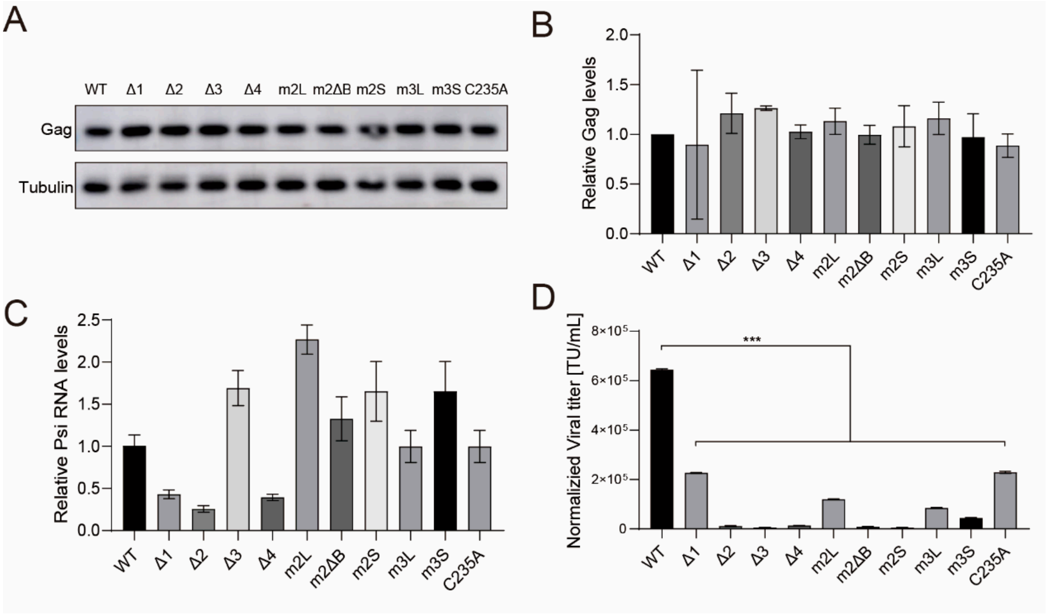

Figure S4. Quantification of Gag protein and viral RNA in pseudovirus-producing HEK293T cells. (A) Western blot analysis of Gag in producer cells co-transfected with psPAX2-EIAVGagpol, pMD2.G, and WT or mutant transfer vectors, probed with anti-Gag antibody; Tubulin serves as a loading control. The single band corresponds to the $Pr55^{gag}$ precursor. (B) Quantification of Gag protein levels in producer cells. Band intensities were measured with ImageJ software, normalized to Tubulin, and presented relative to WT. (C) Relative Psi RNA levels in producer cells. Total RNA was extracted at 48 h post-transfection, and Psi RNA was quantified by RT-qPCR. Data were normalized to *gapdh* and presented relative to WT. (D) Infectious pseudovirus titers normalized to Psi RNA levels. Normalized titers were calculated by dividing infectious titers (from Figure 3C) by Psi RNA levels. Data are mean ± SD (n=3). ***, $p < 0.001$.

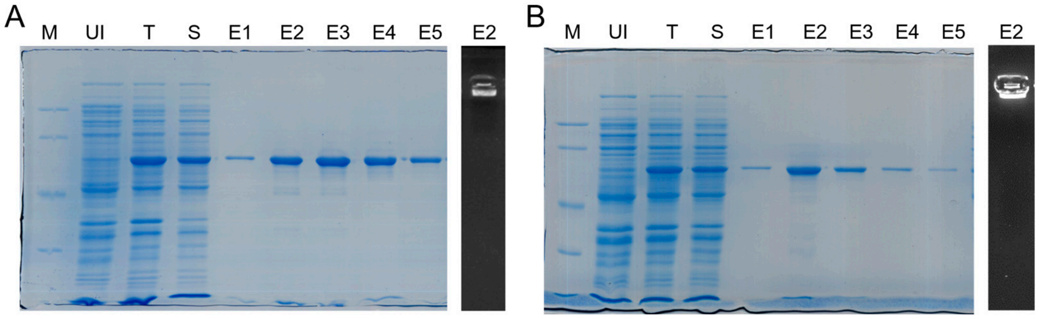

Figure S5. His-tagged protein-RNA complexes purified from *E. coli* expressing Gag (A) or mGag (B) together with EIAV Psi. (A, B) Left panels: SDS-PAGE analysis of Ni-NTA affinity purification fractions. M, protein molecular weight marker (from top to bottom: 97.2, 66.7, 44.3, 29.0 kDa); UI, uninduced whole cell lysate; T, total cell lysate; S, soluble fraction; FT, flow-through; E1–E5, elution fractions 1–5. Proteins were resolved on 10% SDS-PAGE gels and stained with Coomassie Brilliant Blue. (A, B) Right panels: Agarose gel analysis of co-purified RNA from elution fraction E2 visualized by ethidium bromide staining.

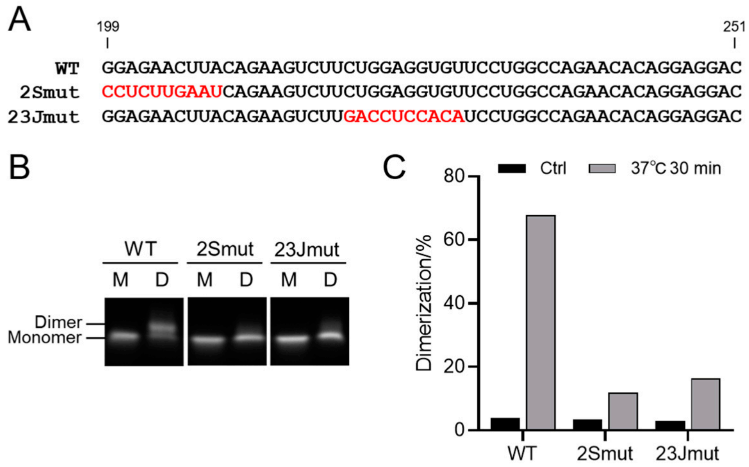

Figure S6. Functional analysis of 2S and 23J in Psi RNA dimerization. (A) Nucleotide sequence of the WT, 2Smut, and 23Jmut. Mutated nucleotides are highlighted in red. (B) WT, 2Smut, and 23Jmut RNAs were incubated in monomerization (M) or dimerization (D) buffer, then analyzed by electrophoresis on 1% agarose gels in TBM buffer at 4°C. (C) Quantification of RNA dimerization efficiency. Dimer percentages were calculated from band intensities in (B) using ImageJ software.

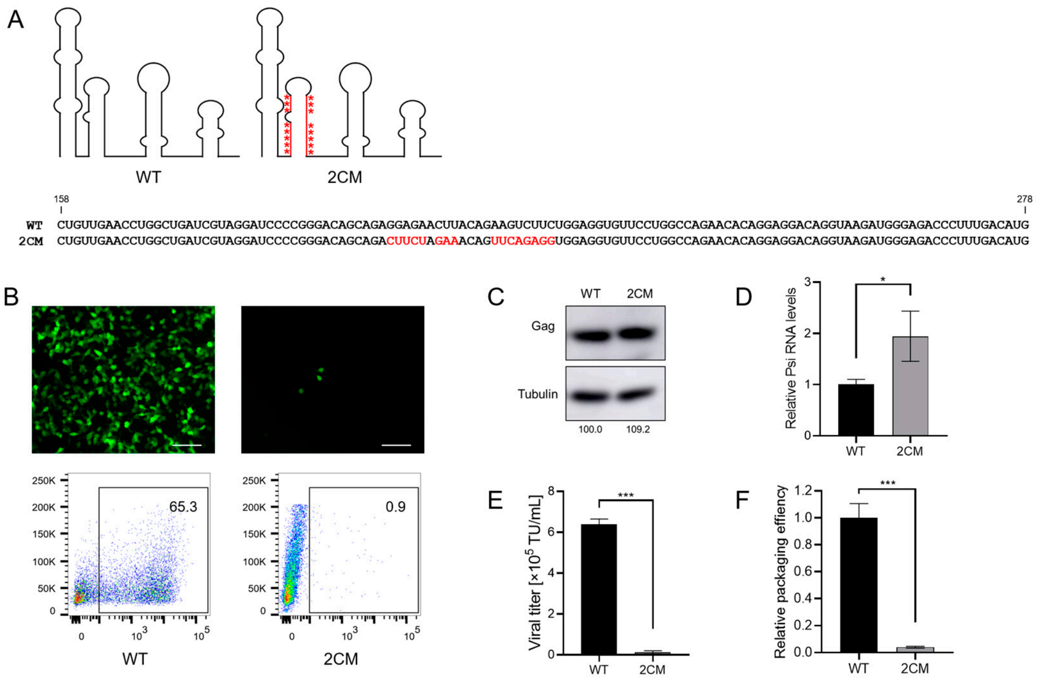

Figure S7. Compensatory mutational analysis of EIAV Psi SL2 in viral packaging and replication. (A) Schematic of WT and compensatory mutant RNA construct. Red asterisks indicate substitutions. Detailed sequences are provided in the lower part. Mutated nucleotides are highlighted in red. (B) Pseudovirus packaging efficiency assay. HEK293T cells (2×10^5) were infected with 200 μL of WT or mutant pseudovirus supernatants. EGFP expression was analyzed at 72 h post-infection. Top: Representative fluorescence microscopy images. Scale bar, 100 μm. Bottom: Flow cytometry dot plots. Boxed regions indicate EGFP-positive populations, with percentages shown. (C) Western blot analysis of Gag in producer cells co-transfected with psPAX2-EIAVGagpol, pMD2.G, and WT or mutant transfer vectors, probed with anti-Gag antibody; Tubulin serves as a loading control. Numbers indicate Gag protein expression relative to control. (D) Relative Psi RNA levels in producer cells. Total RNA was extracted at 48 h post-transfection, and Psi RNA was quantified by RT-qPCR. Data were normalized to *gapdh* and presented relative to WT. (E) Infectious titers of WT and Psi mutant pseudoviruses, quantified based on the data in (B). (F) Relative RNA packaging efficiency of WT and mutant viruses. Packaging efficiency was calculated as the ratio of virion-associated Psi RNA to intracellular Psi RNA, with wild-type set as 1.0. Data are mean ± SD (n=3). *, $0.01 < p < 0.05$; ***, $p < 0.001$.

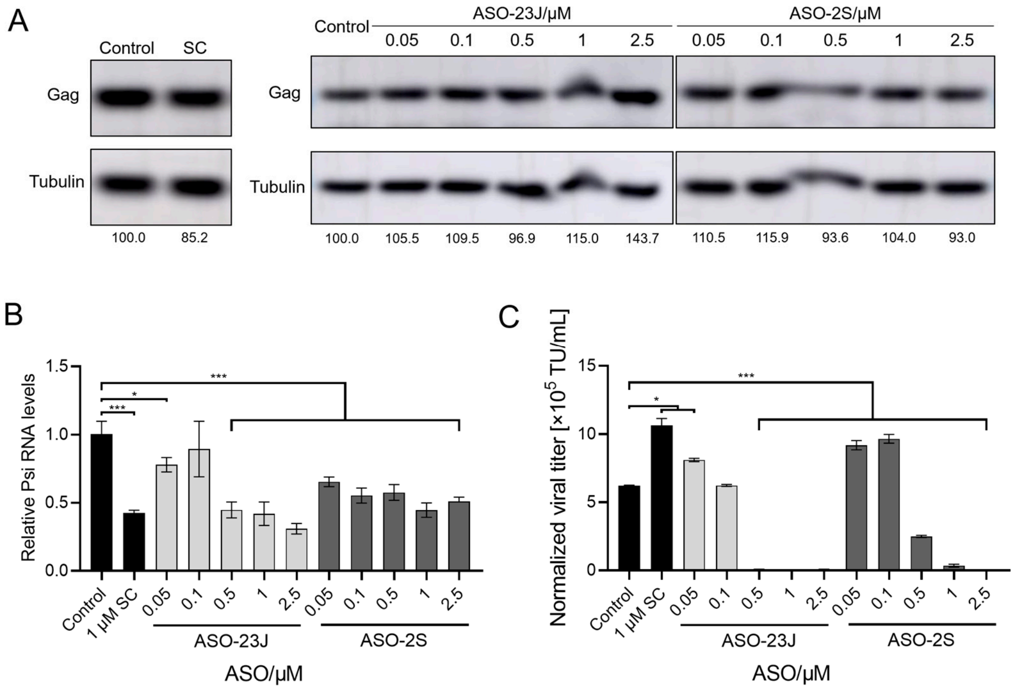

Figure S8. Quantification of Gag protein and viral RNA in ASO-treated pseudovirus-producing HEK293T cells. (A) Western blot analysis of Gag in wild-type pseudovirus-producing cells treated with the indicated ASO concentrations, probed with anti-Gag antibody; Tubulin serves as a loading control. Numbers indicate Gag protein expression relative to control. (B) Relative Psi RNA levels in producer cells. Total RNA was extracted at 48 h post-transfection, and Psi RNA was quantified by RT-qPCR. Data were normalized to *gapdh* and presented relative to WT. (C) Infectious pseudovirus titers normalized to Psi RNA levels. Normalized titers were calculated by dividing infectious titers (from Figure 5B and F) by Psi RNA levels. Data are mean ± SD (n=3). *, $0.01 < p < 0.05$; ***, $p < 0.001$.

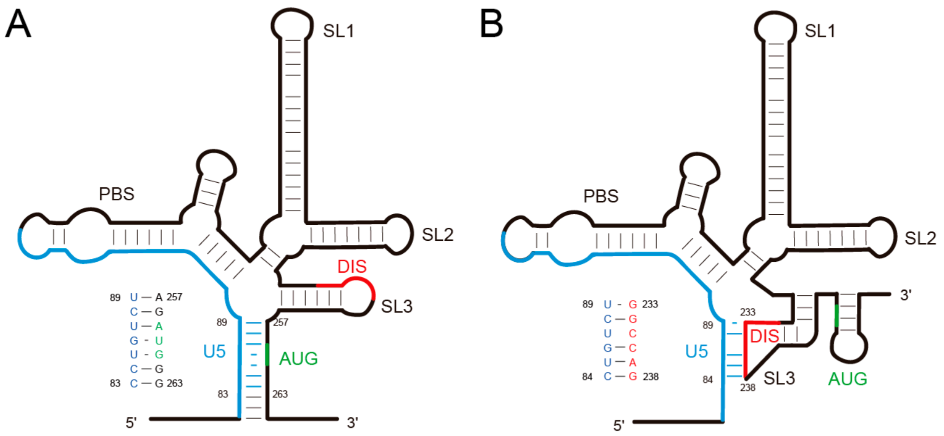

Figure S9. Hypothetical conformational switch models for the EIAV 5′ leader. (A) Putative packaging-competent conformation. The U5 region (blue) pairs with the AUG (green), leaving the palindromic sequence in SL3 (red) exposed. (B) Putative translation-competent conformation. U5 is predicted to dissociate from the AUG and pair with the palindromic region in SL3, potentially facilitating Gag translation. Insets show nucleotide details of U5-AUG and U5-palindrome base pairing.

Table S1. Primers used in this study.

Primer	Sequence (5' to 3')
Primers for DMS modification	
DMS-F	TGGGCTGAAAAGGCCTTTGT
DMS-R	CTACTAGACTTAGCGCCCAA
Primers for plasmid construction	
CMV-F1	ACTACAAACTTAGTAGTACGCGTTGACATTGATTATTGACTAGTTATT
CMV-R1	TGCCCTCGAGGCGCTGCTTATATAGACCTCCCACCGTACA
RU5-F	CAGCGCCTCGAGGGCACTCAGATTCTGCGGTCT
RU5-R	CAATACCCTGCAGCAGCGTCTGAGTTACATCTTCCAGCAATG
cPPT-F	ACGCTGCTGCAGGGTATTGTAGAAAGGGCAAATAG
cPPT-R	ATGAATTCTGCAACAATACTCAATTTGGCTTTA
CMV-F2	GCTCTAGATAGCCCATATATGGAGTTCCGCGTTACA
EGFP-R	ATATCGATTTACTTGTACAGCTCGTCCATGCCGAGAG
dP-F	AGGTAAGATGGGAGACCCTTTGAC
dP-R	GGGTCTCCCATCTTACCTCCCGGGCTGCAGGAAT
Psi-F	AAGCCTCGAGTTCTCTACTCAGTCCCTGTC
Psi-R	GCTAAAGCTTGCTCCATGTCAAAGGGTCT
Primers for mutant construction	
Δ1-F	AGGAGAACTTACAGAAGTCTTCTGGAGGTG
Δ1-R	GTAGGGTCTGCGCCCCTCTC
Δ2-F	GGAGGTGTTCCTGGCCAGAACA
Δ2-R	CTGCTGTCCCGGGGATCCTAC
Δ3-F	AGGTAAGATGGGAGACCCTTTGAC
Δ3-R	CTCCAGAAGACTTCTGTAAGTTCTCCT
Δ4-F	GACATGGAGCAAGGCGCTCAA
Δ4-R	TACCTGTCCTCCTGTGTTCTGG
m2S-F	TCTTCTGAAACAGAAGTCTTCTGGAGGTGTTCCT
ΔB-F	AGAAGACTTACAGAAGTCTTCTGGAGGTGTTCCT
m2L-F	AGGAGAACTTTTCGAAGTCTTCTGGAGGTGTTCCTG
m3S-F	GACGCCAGAACACAGGAGGACAG
m3S-R	CTCCACCTCCAGAAGACTTCTGTAAGTT
m3L-F	AGGTGCAGGAGGACAGGTAAGATGGGAGA
m3L-R	CAATCAGGAACACCTCCAGAAGACTTCTGTAAGT
C235A-F	TGGACAGAACACAGGAGGACAGGTA
C235A-R	GGAACACCTCCAGAAGACTTCTGTA
2CM-F	CTTCTAGAAACAGTTCAGAGGTGGAGGTGTTCCTGGCCAGAA
2CM-R	CCACCTCTGAACTGTTTCTAGAAGTCTGCTGTCCCGGGGATC
2Smut-F	GACCTCCACATCCTGGCCAGAACACAGGAG
2Smut-R	GGCCAGGATGTGGAGGTCAAGACTTCTGTAAGTTCTCCTCTG
23Jmut-F	GCAGACCTCTTGAATCAGAAGTCTTCTGGAGGTGTTCCT
23Jmut-R	GACTTCTGATTCAAGAGGTCTGCTGTCCCGGGGAT
Primers for dimerization assay	
M13F(-47)	CGCCAGGGTTTTCCCAGTCACGAC
dimer-R	CCTTCTCTAACTTCTTGAGCGCCTTG
Primers for RT-qPCR	
qPsi-F	TGAGAGGGGCGCAGACCCTA
qPsi-R	CCTTCTCTAACTTCTTGAGCGCCTTG
GAPDH-F	GAGTCCACTGGCGTCTTCAC
GAPDH-R	ATGACGAACATGGGGGCATC
Primers for SG-PERT	
MS2-qF	CCACGCAGCCAATCAGAGTC
MS2-qR	GGGAGAGTGTGGTTTGTGTAAGAAT

Table S2. Plasmids used in this study.

Plasmid	Description
EIAVuk	EIAV infectious clone
pBS-RU5	*In vitro* transcription template harboring WT EIAV 5' leader
pBS-RU5-Δ1	*In vitro* transcription template harboring SL1-deleted EIAV 5' leader
pBS-RU5-Δ3	*In vitro* transcription template harboring SL3-deleted EIAV 5' leader
pBS-RU5-m3S	*In vitro* transcription template harboring SL3 stem-mutated EIAV 5' leader
pBS-RU5-m3L	*In vitro* transcription template harboring SL3 loop-mutated EIAV 5' leader
pBS-RU5-C235A	*In vitro* transcription template harboring 5' leader with C235A substitution
pBS-RU5-2Smut	*In vitro* transcription template harboring 2S-mutated EIAV 5' leader
pBS-RU5-23Jmut	*In vitro* transcription template harboring 23J-mutated EIAV 5' leader
pMD2.G	VSV-G envelope expressing plasmid
psPAX2	Lentiviral packaging plasmid
psPAX2-EIAVGagpol	EIAV packaging plasmid
pEIAV	EIAV transfer plasmid
pEIAV-CEGFP	EIAV transfer plasmid expressing EGFP
pEIAV-Δ1-CEGFP	EIAV transfer plasmid harboring SL1 deletion and expressing EGFP
pEIAV-Δ2-CEGFP	EIAV transfer plasmid harboring SL2 deletion and expressing EGFP
pEIAV-Δ3-CEGFP	EIAV transfer plasmid harboring SL3 deletion and expressing EGFP
pEIAV-Δ4-CEGFP	EIAV transfer plasmid harboring SL4 deletion and expressing EGFP
pEIAV-m2L-CEGFP	EIAV transfer plasmid harboring SL2 loop mutation and expressing EGFP
pEIAV-m2ΔB-CEGFP	EIAV transfer plasmid harboring SL2 bulge repair and expressing EGFP
pEIAV-m2S-CEGFP	EIAV transfer plasmid harboring SL2 stem mutation and expressing EGFP
pEIAV-m3L-CEGFP	EIAV transfer plasmid harboring SL3 loop mutation and expressing EGFP
pEIAV-m3S-CEGFP	EIAV transfer plasmid harboring SL3 stem mutation and expressing EGFP
pEIAV-C235A-CEGFP	EIAV transfer plasmid harboring C235A substitution and expressing EGFP
pEIAV-2CM-CEGFP	EIAV transfer plasmid harboring SL2 compensatory mutation and expressing EGFP
EIAVuk-Δ1	EIAV infectious clone with SL1 deletion
EIAVuk-Δ2	EIAV infectious clone with SL2 deletion
EIAVuk-Δ3	EIAV infectious clone with SL3 deletion
EIAVuk-m2L	EIAV infectious clone with SL2 loop mutation
EIAVuk-m2ΔB	EIAV infectious clone with repaired SL2 bulge
EIAVuk-m2S	EIAV infectious clone with SL2 stem mutation
EIAVuk-m3L	EIAV infectious clone with SL3 loop mutation
EIAVuk-m3S	EIAV infectious clone with SL3 stem mutation
EIAVuk-C235A	EIAV infectious clone with C235A substitution
pBS-Psi	pBS plasmid for expressing EIAV Psi in *E. coli*
pET28b-EIAV Gag	pET28 plasmid for expressing EIAV Gag in *E. coli*
pET28b-EIAV mGag	pET28 plasmid for expressing EIAV GagH391K/H410K in *E. coli*
